# Diagnostic value of DECT-based colored collagen maps for the assessment of cruciate ligaments in patients with acute trauma

**DOI:** 10.1007/s00330-023-09558-4

**Published:** 2023-03-31

**Authors:** Leon D. Gruenewald, Vitali Koch, Simon S. Martin, Ibrahim Yel, Scherwin Mahmoudi, Simon Bernatz, Katrin Eichler, Leona S. Alizadeh, Tommaso D’Angelo, Silvio Mazziotti, Hendrik Singer, Vincent Heck, Thomas J. Vogl, Christian Booz

**Affiliations:** 1 Division of Experimental Imaging, Department of Diagnostic and Interventional Radiology, University Hospital Frankfurt, Theodor-Stern-Kai 7, 60590 Frankfurt Am Main, Germany; 2 Department of Diagnostic and Interventional Radiology, University Hospital Frankfurt, Frankfurt Am Main, Germany; 3 Department of Biomedical Sciences and Morphological and Functional Imaging, University Hospital Messina, Messina, Italy

**Keywords:** Multidetector computed tomography, Knee joint, Collagen, Anterior cruciate ligament, Posterior cruciate ligament

## Abstract

**Objectives:**

The purpose of this study was to evaluate the diagnostic accuracy of third-generation dual-source dual-energy CT (DECT) color-coded collagen reconstructions for the assessment of the cruciate ligaments compared to standard grayscale image reconstruction.

**Methods:**

Patients who underwent third-generation dual-source DECT followed by either 3-T MRI or arthroscopy of the knee joint within 14 days between January 2016 and December 2021 were included in this retrospective study. Five radiologists independently evaluated conventional grayscale DECT for the presence of injury to the cruciate ligaments; after 4 weeks, readers re-evaluated the examinations using grayscale images and color-coded collagen reconstructions. A reference standard for MRI was provided by a consensus reading of two experienced readers and arthroscopy. Sensitivity and specificity were the primary metrics of diagnostic performance.

**Results:**

Eighty-five patients (mean age, 44 years ± 16; 50 male) with injury to the ACL or PCL (*n* = 31) were ultimately included. Color-coded collagen reconstructions significantly increased overall sensitivity (94/105 [90%] vs. 67/105 [64%]), specificity (248/320 [78%] vs. 215/320 [67%]), PPV (94/166 [57%] vs. 67/162 [39%]), NPV (248/259 [96%] vs. 215/253 [85%]), and accuracy (342/425 [81%] vs. 282/425 [66%]) for the detection of injury to the anterior cruciate ligament (all parameters, *p* < .001). For injury to the posterior cruciate ligament, diagnostic accuracy increased for complete tears (*p* < .001). Color-coded collagen reconstructions achieved superior diagnostic confidence, image quality, and noise scores compared to grayscale CT (all parameters, *p* < .001) and showed good agreement with MRI examinations.

**Conclusions:**

DECT-derived color-coded collagen reconstructions yield substantially higher diagnostic accuracy and confidence for assessing the integrity of the cruciate ligaments compared to standard grayscale CT in patients with acute trauma.

**Key Points:**

• *Color-coded collagen reconstructions derived from dual-energy CT yield substantially higher diagnostic accuracy and confidence for the assessment of the cruciate ligaments compared to standard grayscale CT in patients with acute trauma.*

• *Color-coded collagen reconstructions demonstrate good agreement with MRI for the assessment cruciate ligament injury.*

• *Dual-energy CT may serve as a readily available screening approach for patients with acute trauma to the knee when injury to the cruciate ligaments is suspected.*

**Supplementary Information:**

The online version contains supplementary material available at 10.1007/s00330-023-09558-4.

## Introduction

The cruciate ligaments are major stabilizers of the knee joint. They prevent excessive anterior and posterior translation and restrain tibial rotation, varus stress, and valgus stress [1]. Injury to the anterior cruciate ligament (ACL) is common, with reported incidences of up to 1147 per 100,000 in the general population, and higher incidences among athletes [2, 3]. Isolated injury to the posterior cruciate ligament (PCL) is relatively rare [4].

Cruciate ligament injury can be managed surgically and nonoperatively. In younger patients and athletes, surgical reconstruction of injured cruciate ligaments is generally performed to enable continued high levels of physical activity. Early reconstruction of the ACL reduces the risk of subsequent injury to other soft tissue structures of the knee and improves long-term knee motion compared to delayed reconstruction [5–8]. Furthermore, early ACL reconstruction reduces the time before patients can return to full physical activity, which reduces associated socioeconomic burdens [9].

MRI is the reference standard for imaging of the cruciate ligaments [10]. However, availability of MRI is usually limited during on-call times, which can delay adequate treatment and recovery. Furthermore, MRI is not available for certain patients due to contraindications. CT is readily available to assess musculoskeletal trauma and usually performed in case of complex injuries to the knee or when conventional radiographs do not demonstrate pathology but injury to the knee joint is suspected. In this context, single-energy CT has been shown to provide insufficient soft-tissue contrast and resolution for assessing injury to the cruciate ligaments [11].

Material differentiation and collagen mapping in dual-energy CT (DECT) increase the quality of soft tissue visualization and provide novel information for different musculoskeletal applications compared to conventional single-energy CT [12–18]. Increased diagnostic value of second-generation dual-source grayscale DECT over conventional single-energy CT for assessing the cruciate ligaments has been demonstrated in initial pilot studies that used a simplified approach, with restrictions to complete tears of the cruciate ligaments and an emphasis on monochromatic images [19, 20]. With the advent of third-generation dual-source DECT, increased spatial resolution and advancements in material differentiation have further improved the possibilities for the assessment of soft tissue structures [21, 22]. In this context, a novel postprocessing algorithm visualizing collagenous structures by application of dedicated material decomposition has been developed [17]. In contrast to most DECT applications, this algorithm does not identify collagen based on the atomic number of contained hydrogen, carbon, nitrogen, and oxygen, but rather on the characteristic attenuation of the densely packed hydroxylysine and hydroxyproline in the collagen side chains, which can be decomposed against water and soft tissue [23, 24]. However, this algorithm has not yet been investigated for evaluation of the cruciate ligaments. Therefore, the purpose of this study was to evaluate the diagnostic accuracy and reader confidence of third-generation dual-source DECT color-coded collagen reconstructions for assessing the cruciate ligaments compared to standard grayscale CT.

## Materials and methods

The institutional review board approved this retrospective study. The requirement to obtain written informed consent was waived. C.B. and I.Y. received speaking fees from Siemens Healthineers. The other authors have no conflict of interest to disclose.

### Patient selection

One hundred consecutive patients over the age of 18 years with acute injury (within 3 days of trauma) to the knee who had undergone non-contrast third-generation dual-source DECT between January 2016 and December 2021, followed by supplementary MRI and/or arthroscopic inspection of the knee joint within 14 days, were considered for study inclusion. Exclusion criteria were suspected or known malignancy, inflammatory conditions, and inadequate imaging quality due to metallic implants.

### CT protocol

CT studies were performed on a third-generation dual-source CT system in dual-energy mode (SOMATOM Force; Siemens Healthineers). Both X-ray tubes operated at different kilovoltage settings (tube A: 90 kVp, 180 mAs; tube B: Sn150 kVp [0.64-mm tin filter], 180 mAs). All examinations were performed without the administration of a contrast agent. Three image sets were acquired in each CT examination: 90 kVp, Sn150 kVp, and weighted average (ratio, 0.5:0.5) to resemble single-energy 120 kVp images. Image series (axial, coronal, and sagittal: section thickness 1 mm, increment 0.75 mm) were reconstructed with dedicated dual-energy bone (Br69f) and soft-tissue kernels (Br40). The image series were automatically transferred to the picture archiving and communication system (PACS; GE Healthcare).

### CT postprocessing

Color-coded collagen reconstruction was performed on a Siemens *syngo.*via VB50 (Siemens Healthineers) using an experimental, not publicly available algorithm with the following default settings:

- Application profile = Knee; Settings = Collagen; Public Layout = Fat Map; Width = 63; Level =  − 32.

For image analysis, axial, coronal, and sagittal color-coded reconstructions (section thickness 1 mm, increment 0.75 mm) were sent to the PACS (GE Healthcare). Reconstruction time was measured from launching Siemens *syngo.*via until image export to the PACS system was initiated.

### MRI protocol

MRI was performed on a 3-Tesla system (PrismaFit, Siemens Healthineers). Examinations included non-contrast T1-weighted turbo spin-echo sequences with and without fat suppression and proton density-weighted sequences with and without fat suppression, all in the transversal, sagittal, and coronal planes (ST: 3 mm). Pulse sequence parameters (echo time, repetition time, flip angle), FOV, and acquisition matrix were adapted for every examination.

### Image analysis

Image evaluation was performed with a conventional PACS workstation (Centricity 7.0; GE Healthcare).

Two board-certified radiologists (K.E. and T.J.V.) with 17 and 35 years of experience in musculoskeletal imaging independently performed a reading of all MRI series to provide an independent reference standard. In case of disagreement (*n* = 2), a third board-certified radiologist (T.G.) with 11 years in musculoskeletal imaging was consulted. ACL and PCL were evaluated separately (1 = rupture certainly absent, 2 = total rupture, 3 = partial rupture, 4 = avulsion).

A consecutive reading of all CT images was independently performed by five radiologists (3 board-certified radiologists (C.B., I.Y., and S.M.)) and two radiologists in training (L.G., V.K.) with 1 to 7 years of experience in musculoskeletal imaging. These readers were blinded to clinical data, imaging results, and follow-up examinations. Two protocols were provided for the assessment of the cruciate ligaments: protocol 1 = standard grayscale images in axial, coronal, and sagittal plane; and protocol 2 = standard grayscale images and color-coded collagen reconstructions in axial, coronal, and sagittal plane as described above. Preset window settings could be freely modified. To reduce observer recall bias, a time interval of 4 weeks was kept between readout sessions and all images were presented in random order for both readouts. Readers reviewed both protocols for the presence of injury to the ACL and PCL (1 = rupture certainly absent, 2 = total rupture, 3 = partial rupture, 4 = avulsion).

Furthermore, readers rated their overall diagnostic confidence in the assessment of cruciate ligament injury as well as the image quality (including soft-tissue contrast, resolution, and artifacts) and image noise (ranging from 1 = poor to 5 = excellent) for each imaging protocol and patient.

### Arthroscopy

Arthroscopy was performed by two board-certified orthopedic surgeons (V.H. and K.Z.) with 8 and 17 years of experience.

### Statistical analysis

Statistical analysis was performed with dedicated commercial software (Prism 9 for macOS, version 9.0.1, GraphPad Software LLC; MedCalc for Windows, Version 20.022, MedCalc). Differences in baseline characteristics were assessed using *t*-tests, if applicable, or Chi-squared tests. Inter-reader agreement was evaluated by computing weighted Fleiss’ *κ*. Agreement between MRI and both imaging protocols was evaluated by computing Cohens’ *κ*.

Imaging findings were analyzed individually for each type of injury to the cruciate ligament, as mentioned above. Furthermore, analysis was performed after lesions were dichotomized (0 = injury absent, 1 = injury present). Findings were compiled in cross-tables, and diagnostic accuracy parameters (sensitivity, specificity, positive predictive value (PPV), negative predictive value (NPV), and area under the curve (AUC)) for the detection of injury to the cruciate ligaments were calculated. Receiver operator characteristic (ROC) curve comparison was used to determine the incremental diagnostic value of color-coding over grayscale imaging. Statistical significance was given if *p* < 0.05.

## Results

**Fig. 1:**
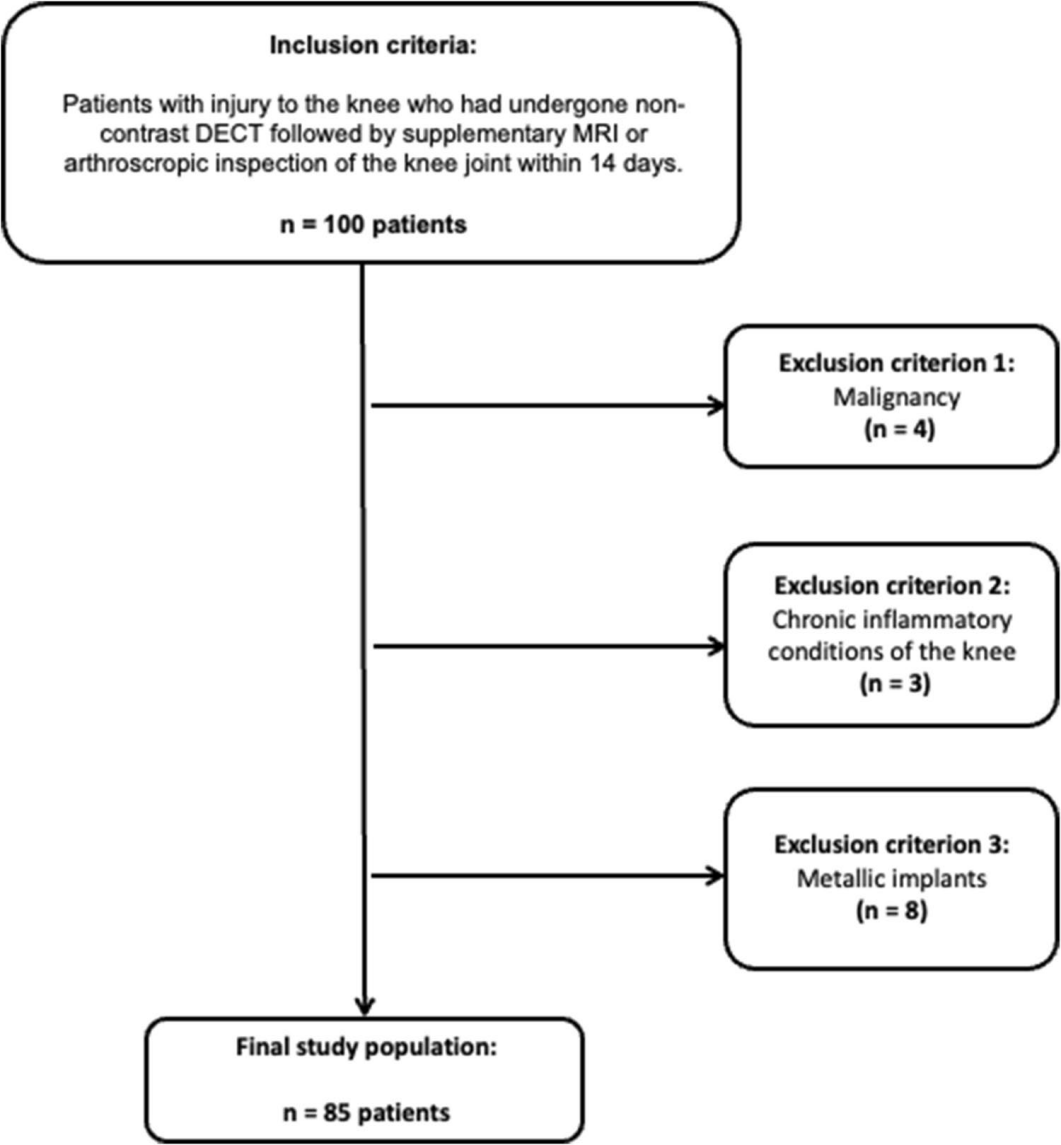
STARD (Standards for Reporting of Diagnostic Accuracy Studies) flowchart of patient inclusion

**Fig. 2:**
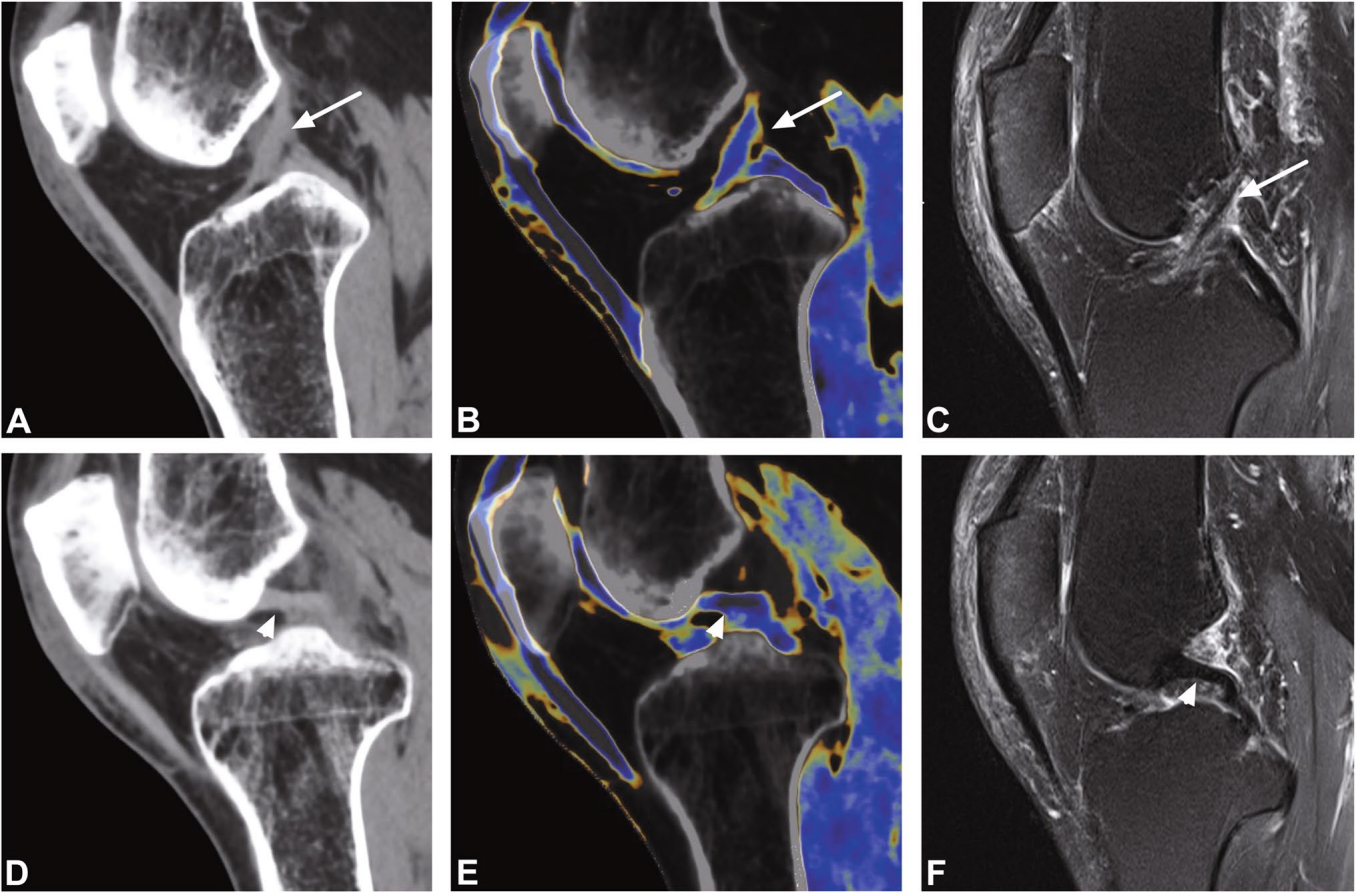
Standard sagittal unenhanced grayscale CT (**A**, **D**), color-coded collagen reconstructions (**B**, **E**), and unenhanced proton density-weighted MRI series with fat saturation (**C**, **F**) of a 28-year-old patient with acute knee pain following a bicycle accident demonstrate intact anterior (arrows) and posterior cruciate ligaments (arrowheads). The collagen signal is visualized blue by using default postprocessing settings

Of 100 patients considered for study inclusion, 15 patients were excluded due to suspected or known malignancy (*n* = 4), inflammatory conditions (*n* = 3), and inadequate imaging quality due to metallic implants (*n* = 8) (Fig. 1). Therefore, a total of 85 patients who had undergone non-contrast third-generation dual-source DECT of the knee joint followed by MRI (*n* = 78) or arthroscopic inspection (*n* = 34) were finally included in this study (50 male and 35 female, mean age, 44 ± 16 years; range, 14–89 years; Table 1). Seven patients did not undergo MRI prior to arthroscopy. Both MRI and arthroscopic inspection were available as a reference standard in 27 patients. In two of these patients, arthroscopy revealed partial rupture of the ACL that were missed in MRI. No lesions of the PCL were missed in MRI. Together, the reference standards revealed an injury to the ACL in 21 patients (24.7%; complete tear = 11; partial tear = 4; avulsion fracture = 6) and the PCL in 10 patients (12.9%; complete tear = 1; partial tear = 0; avulsion fracture = 9). One patient had injury to the ACL and PCL. Using only MRI as the reference standard revealed injury to the ACL in 19 patients (complete tear = 11; partial tear = 2; avulsion = 6) and to the PCL in 10 patients (complete tear = 1; avulsion fracture = 9). No statistical significance was observed between the demographics of patients with injury to the cruciate ligaments and patients without injury. Patient characteristics are summarized in Table 1. The mean interval between dual-energy CT and MRI or arthroscopic inspection was 6 days (range, 0–13 days). Color-coded image reconstruction took on average 3 min (range, 1–5 min). An example case without injury to the cruciate ligaments is given in Fig. 2.

**Table 1:** Characterization of the patient population

Patient characteristics	Cruciate ligament injury (*n* = 30)	No cruciate ligament injury (*n* = 55)	*p* value
Age (years)	46.4 ± 15.3	43.5 ± 16.4	0.61
Sex (*n*)			
Male	18 (60.0%)	32 (58.2%)	0.88
Female	12 (40.0%)	23 (41.8%)	0.88
ACL injury	*n* = 21		
• Complete tear	*n* = 11 (52.4%)		
• Partial tear	*n* = 4 (19.0%)		
• Avulsion fracture	*n* = 6 (28.6%)		
PCL injury	*n* = 10		
• Complete tear	*n* = 1 (10.0%)		
• Partial tear	*n* = 0 (0.0%)		
• Avulsion fracture	*n* = 9 (90.0%)		

*Abbreviations*: *ACL*, anterior cruciate ligament; *PCL*, posterior cruciate ligament

Age is given ± standard deviation (*SD*)

No significant differences were observed between the demographics of patients with injury to the cruciate ligaments and patients without cruciate ligament injury. One patient had injury to the ACL and PCL

### Diagnostic accuracy of ACL injury

**Fig. 3:**
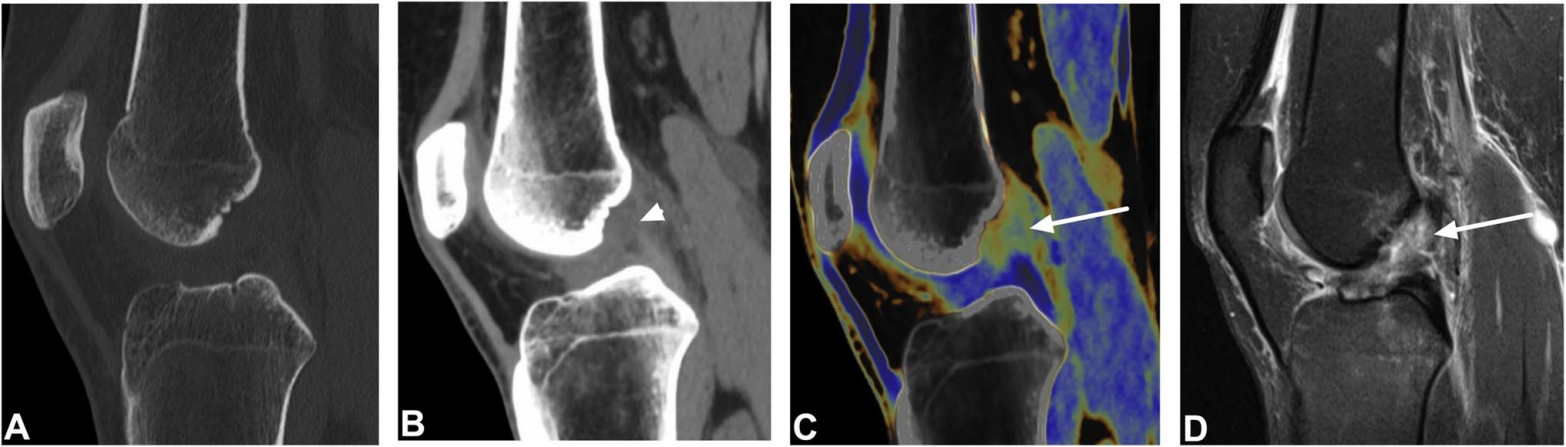
Standard sagittal unenhanced grayscale CT (**A**, **B**), color-coded collagen reconstructions (**C**), and proton density-weighted unenhanced MRI series with fat saturation (**D**) of a complete tear (arrows) of the anterior cruciate ligament (ACL) in the cranial part in a 31-year-old professional kickboxer who repeatedly twisted her knee during kickboxing and presented to the emergency department with knee pain on Friday night immediately after training. Standard grayscale CT images showed no presence of fracture. However, a subtle inhomogeneity of the ACL in the cranial aspect (arrowhead) was noted. Color-coded collagen maps showed a distinct lack of collagen signal in this area (arrow) indicating a complete tear of the ACL. Subsequently, the additionally performed MRI confirmed the diagnosis, which was treated the next morning by arthroscopic repair. Abbreviations: ACL, anterior cruciate ligament

**Fig. 4:**
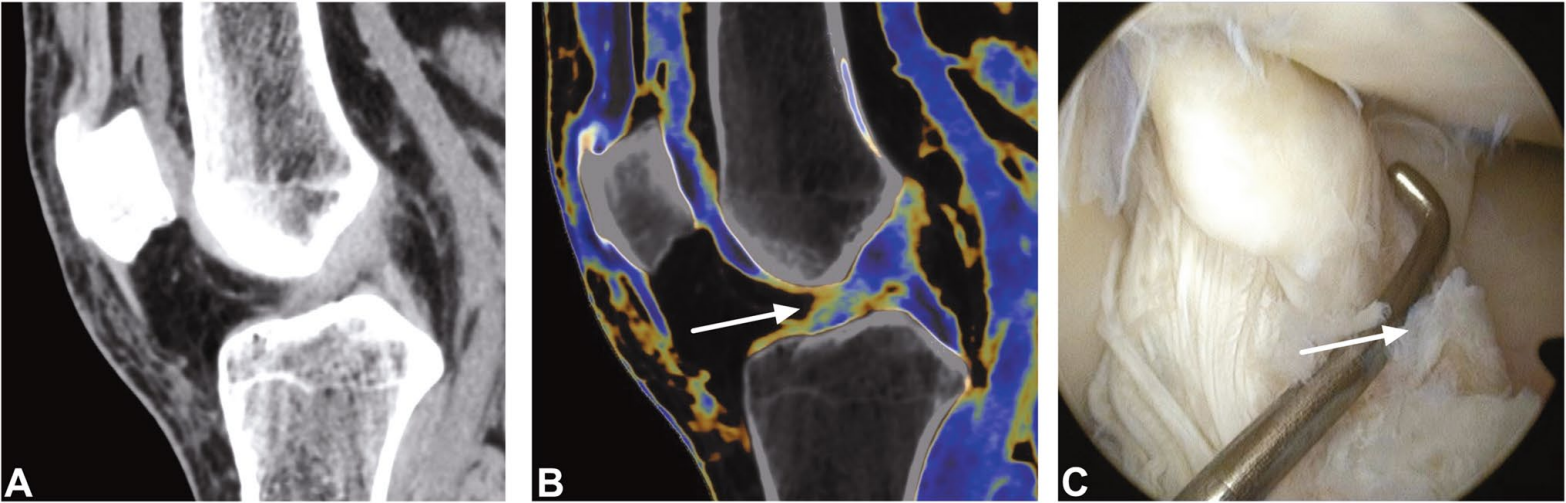
Standard sagittal unenhanced grayscale CT, color-coded collagen reconstructions, and arthroscopy images of a partial tear of the anterior cruciate ligament (ACL) in a 23-year-old patient who twisted his knee playing football. The ACL was considered as completely intact by the radiologists in this study using the standard grayscale CT (**A**). However, color-coded collagen reconstruction maps showed reduced and thinned blue collagen signal close to the tibial insertion (arrow) in terms of a partial tear (**B**), which was confirmed by the subsequently performed arthroscopy (arrow) (**C**). Abbreviations: ACL, anterior cruciate ligament

Protocol 2 including color-coded collagen reconstructions of the ACL showed higher overall sensitivity (94/105 [90%] vs. 67/105 [64%]), specificity (248/320 [78%] vs. 215/320 [67%]), PPV (94/166 [57%] vs. 67/162 [39%]), NPV (248/259 [96%] vs. 215/253 [85%]), accuracy (342/425 [81%] vs. 282/425 [66%]), and AUC (0.84 vs 0.66) for the detection of injury to the ACL compared to protocol 1 comprising standard grayscale CT images alone (all comparisons, ΔAUC = 0.18, *p* < 0.001, Table 2). Inter-reader agreement was excellent for both protocols (*κ* = 0.84 for protocol 1 and *κ* = 0.85 for protocol 2), but only moderate between protocol 1 and protocol 2 (*κ* = 0.52). Notably, increases in AUC between protocol 1 and protocol 2 were highest for complete ACL tears (0.85 vs. 0.52, ΔAUC = 0.33, *p* < 0.001) and partial ACL tears (0.75 vs. 0.53, ΔAUC = 0.22, *p* = 0.02), whereas no significant difference was observed for avulsions of the ACL (0.68 vs. 0.63, ΔAUC = 0.05, *p* = 0.14). Diagnostic accuracy parameters did not change significantly when arthroscopy was excluded as a reference standard. Agreement between MRI and protocol 2, including color-coded collagen reconstructions, was higher compared to agreement between MRI and protocol 1 for all injuries (Table 3). Example cases demonstrating improvement in the detection of complete and partial ACL tears by color-coded collagen reconstructions are illustrated in Figs. 3 and 4.

**Table 2:** Diagnostic accuracy of standard CT and color-coded collagen reconstructions for the ACL and PCL

ACL injury (*n* = 21)	Sensitivity	Specificity	PPV	NPV	Accuracy	AUC	*p* value
Total							
Protocol 1	67/105 (64%) [82–95%]	215/320 (67%) [62–72%]	67/172 (39%) [34–44%]	215/253 (85.0%) [81–88%]	282/425 (66%) [62–71%]	0.66 [0.61–0.70]	< .001
Protocol 2	94/105 (90%) [82–95%]	248/320 (78%) [78–82%]	94/166 (57%) [51–62%]	248/259 (96%) [93–96%]	342/425 (80%) [76–84%]	0.83 [0.80–0.87]	< .001
Complete tear (*n* = 11)							
Protocol 1	5/55 (9%) [3–20%]	354/370 (96%) [93–98%]	5/21 (24%) [11–45%]	354/404 (88%) [87–89%]	359/425 (84%) [81–88%]	0.52 [0.48–0.57]	< .001
Protocol 2	41/55 (75%) [61–85%]	350/370 (95%) [92–97%]	41/61 (67%) [57–76%]	350/364 (96%) [94–98%]	391/425 (92%) [89–94%]	0.85 [0.81–0.88]	< .001
Partial tear (*n* = 4)							
Protocol 1	7/20 (35%) [15–59%]	290/405 (72%) [67–76%]	7/122 (6%) [3–10%]	290/303 (96%) [94–97%]	297/425 (70%) [65–74%]	0.53 [0.48–0.58]	0.02
Protocol 2	13/20 (65%) [41–85%]	342/405 (84%) [81–88%]	13/76 (17%) [12–23%]	342/349 (98%) [96–99%]	355/425 (84%) [80–87%]	0.75 [0.70–0.79]	0.02
Avulsion fracture (*n* = 6)							
Protocol 1	9/30 (30%) [15–49%]	375/395 (95%) [92–97%]	9/29 (31%) [18–47%]	375/396 (95%) [93–96%]	384/425 (90%) [87–93%]	0.63 [0.58–0.67]	0.14
Protocol 2	12/30 (40%) [23–59%]	378/395 (96%) [93–97%]	12/29 (41%) [27–57%]	378/396 (95%) [94–97%]	390/425 (92%) [89–94%]	0.68 [0.63–0.72]	0.14
PCL injury (*n* = 10)	Sensitivity	Specificity	PPV	NPV	Accuracy	AUC	*p*-value
Total							
Protocol 1	44/50 (88%) [76–95%]	303/375 (81%) [76–85%]	44/116 (38%) [33–44%]	303/309 (98%) [96–99%]	347/425 (82%) [78–85%]	0.84 [0.81–0.88]	0.20
Protocol 2	42/50 (84%) [71–93%]	355/375 (95%) [92–97%]	42/62 (68%) [57–77%]	355/363 (98%) [97–99%]	397/425 (93%) [91–96%]	0.89 [0.86–0.92]	0.20
Complete tear (*n* = 1)							
Protocol 1	0/5 (0%) [0–52%]	414/420 (99%) [97–99%]	0/6 (0%) [0–0%]	414/419 (99%) [99–99%]	414/425 (97%) [95–99%]	0.49 [0.44–0.54]	0.02
Protocol 2	3/5 (60%) [15–95%]	420/420 (100%) [99–100%]	3/3 (100%) [100–100%]	420/422 (100% [99–100%]	423/425 (100%) [98–100%]	0.80 [0.76–0.837]	0.02
Avulsion fracture (*n* = 9)							
Protocol 1	33/45 (73%) [58–85%]	366/380 (96%) [94–98%]	33/47 (70%) [58–80%]	366/378 (97%) [95–98%]	399/425 (94%) [91–96%]	0.85 [0.81–0.88]	0.81
Protocol 2	32/45 (71%) [56–84%]	369/380 (97%) [95–99%]	32/43 (74%) [61–84%]	369/382 (97%) [95–98%]	401/425 (94%) [92–96%]	0.84 [0.80–0.88]	0.81

*Abbreviations*: *ACL* anterior cruciate ligament, *PCL* posterior cruciate ligament, *PPV* positive predictive value, *NPV* negative predictive value, *AUC* area under the curve

Numbers in square brackets are confidence intervals. Diagnostic accuracy of protocol 1 (standard grayscale CT) and protocol 2 (standard grayscale CT + color-coded collagen reconstructions) with MRI or arthroscopic inspection as the standard of reference

**Table 3:** Diagnostic accuracy of standard CT and color-coded collagen reconstructions for the ACL and PCL using MRI as the reference standard

ACL injury (*n* = 19)	Sensitivity	Specificity	PPV	NPV	Accuracy	AUC	*p* value	k w/MRI
Total (*n* = 19)								
Protocol 1	60/95 (63%) [53–73%]	197/295 (67%) [61–72%]	60/158 (38%) [33–43%]	197/232 (85%) [81–88%]	257/390 (66%) [61–71%]	0.65 [0.60–0.70]	< .001	0.24 [0.15–0.34]
Protocol 2	87/95 (92%) [84–96%]	277/295 (77%) [72–82%]	87/155 (56%) [51–61%]	227/235 (97%) [94–98%]	314/390 (81%) [76–84%]	0.84 [0.80–0.88]	< .001	0.56 [0.48–0.65]
Complete tear (*n* = 11)								
Protocol 1	5/55 (9%) [3–20%]	321/335 (96%) [93–98%]	5/19 (26%) [12–49%]	321/371 (87%) [86–88%]	326/390 (84%) [80–87%]	0.53 [0.47–0.58]	< .001	0.07 [-0.04—0.18]
Protocol 2	41/55 (75%) [61–85%]	320/335 (96%) [93–98%]	41/56 (73%) [62–82%]	320/334 (96%) [94–97%]	351/390 (93%) [90–95%]	0.85 [0.81–0.88]	< .001	0.70 [0.59–0.80]
Partial tear (*n* = 2)								
Protocol 1	1/10 (10%) [0–45%]	270/380 (71%) [66–76%]	1/111 (1%) [0–6%]	270/279 (97%) [96–97%]	271/390 (70%) [65–74%|	0.41 [0.36–0.46]	< .05	–0.03 [-0.07–0.01]
Protocol 2	8/10 (80%) [44–98%]	319/380 (84%) [80–88%]	8/69 (12%) [8–16%]	319/321 (99%) [98–100%]	327/390 (84%) [80–87%]	0.82 [0.78–0.86]	< .05	0.17 [0.06–0.27]
Avulsion fracture (*n* = 6)								
Protocol 1	9/30 (30%) [15–49%]	342/360 (95%) [92–97%)	9/27 (33%) [20–50%]	342/363 (94%) [93–95%]	351/390 (90%) [83–93%]	0.63 [0.58–0.67]	0.15	0.26 [0.10–4.3]
Protocol 2	12/30 (40%) [23–59%]	344/360 (96%) [93–97%]	12/28 (43%) [28–59%]	344/362 (95%) [94–96%]	356/390 (91%) [88–94%]	0.68 [0.63–0.72]	0.15	0.37 [0.20–0.54]
PCL injury (*n* = 10)	Sensitivity	Specificity	PPV	NPV	Accuracy	AUC	*p*-value	k w/MRI
Total (*n* = 10)								
Protocol 1	44/50 (88%) [76–96%]	272/340 (80%) [75–84%]	44/112 (39%) [34–45%]	272/278 (98%) [96–99%]	316/390 (81%) [77–85%]	0.84 [0.80–0.88]	0.18	0.45 [0.35–0.54]
Protocol 2	42/50 (84%) [71–93%]	321/340 (94.4%) [91–97%]	42/61 (69%) [58–78%]	321/329 (98%) [96–99%]	363/390 (93%) [90–95%]	0.89 [0.86–0.92]	0.18	0.72 [0.62–0.82]
Complete tear (*n* = 1)								
Protocol 1	0/5 (0%) [0–52%]	379/385 (98%) [97–99%]	0/6 (0%) [0–0%)	379/384 (99%) [95–99%]	379/390 (97%) [95–99%]	0.49 [0.44–0.54]	< .05	–0.01 [-0.02–0.01]
Protocol 2	3/5 (60%) [15–95%]	385/385 (100%) [99–100%]	3/3 (100%) [100–100%)	385/387 (100%) [99–100%]	388/390 (100%) [98–100%)	0.80 [0.76–0.84]	< .05	0.75 [0.41–1.0]
Avulsion fracture (*n* = 9)								
Protocol 1	33/45 (73%) [58–84%]	332/345 (96%) [94–98%]	33/46 (72%) [59–82%]	332/344 (97%) [95–98%]	365/390 (94%) [91–96%]	0.85 [0.81–0.88]	0.82	0.69 [0.58–0.80]
Protocol 2	32/45 (71%) [56–84%]	335/345 (97%) [95–99%]	32/42 (76%) [63–86%]	335/348 (96%) [94–98%]	367/390 (94%) [91–96%]	0.84 [0.80–0.88]	0.82	0.70 [0.59–0.82]

*Abbreviations*: *ACL* anterior cruciate ligament, *PCL* posterior cruciate ligament, *PPV* positive predictive value, *NPV* negative predictive value, *AUC* area under the curve, *k* Cohen’s Kappa

Numbers in square brackets are confidence intervals. Diagnostic accuracy of protocol 2 (standard grayscale CT + color-coded collagen reconstructions) with MRI as the only reference standard. No significant differences in diagnostic accuracy were observed when arthroscopic inspection was removed as a reference standard. Kappa statistics demonstrate good agreement between color-coded collagen reconstructions and MRI for the assessment of ligamentous injury

Comparing all CT readers, the least experienced radiologists showed a greater improvement in diagnostic accuracy for the detection of ACL injury compared to more experienced readers with higher overall sensitivity (20/21 [95%] vs. 12/21 [57%]), specificity (52/64 [81%] vs. 41/64 [64%]), PPV (20/34 [63%] vs. 12/35 [34%]), NPV (52/51 [98%] vs. 41/50 [82%]), and accuracy (72/85 [85%] vs. 53/85 [62%], all comparisons, *p* < 0.001) (Supplementary Table 1).

### Diagnostic accuracy of PCL injury

**Fig. 5:**
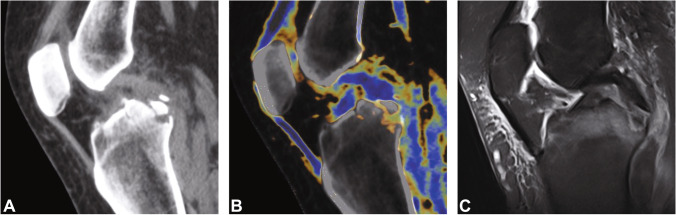
Standard unenhanced grayscale CT, color-coded collagen reconstructions, and unenhanced proton density-weighted MRI series with fat saturation of a 58-year-old patient show a tibial avulsion fracture of the posterior cruciate ligament (PCL) (**A**–**C**, arrows) following a dashboard injury. Color-coded collagen reconstructions and the MRI series demonstrate unambiguously a retained integrity of the thickened PCL (arrowheads), while there is some inhomogeneity of the PCL on standard grayscale CT series which led to a decreased readers’ diagnostic confidence when using protocol 1 compared to protocol 2 in this case. Abbreviations: PCL, posterior cruciate ligament

**Fig. 6:**
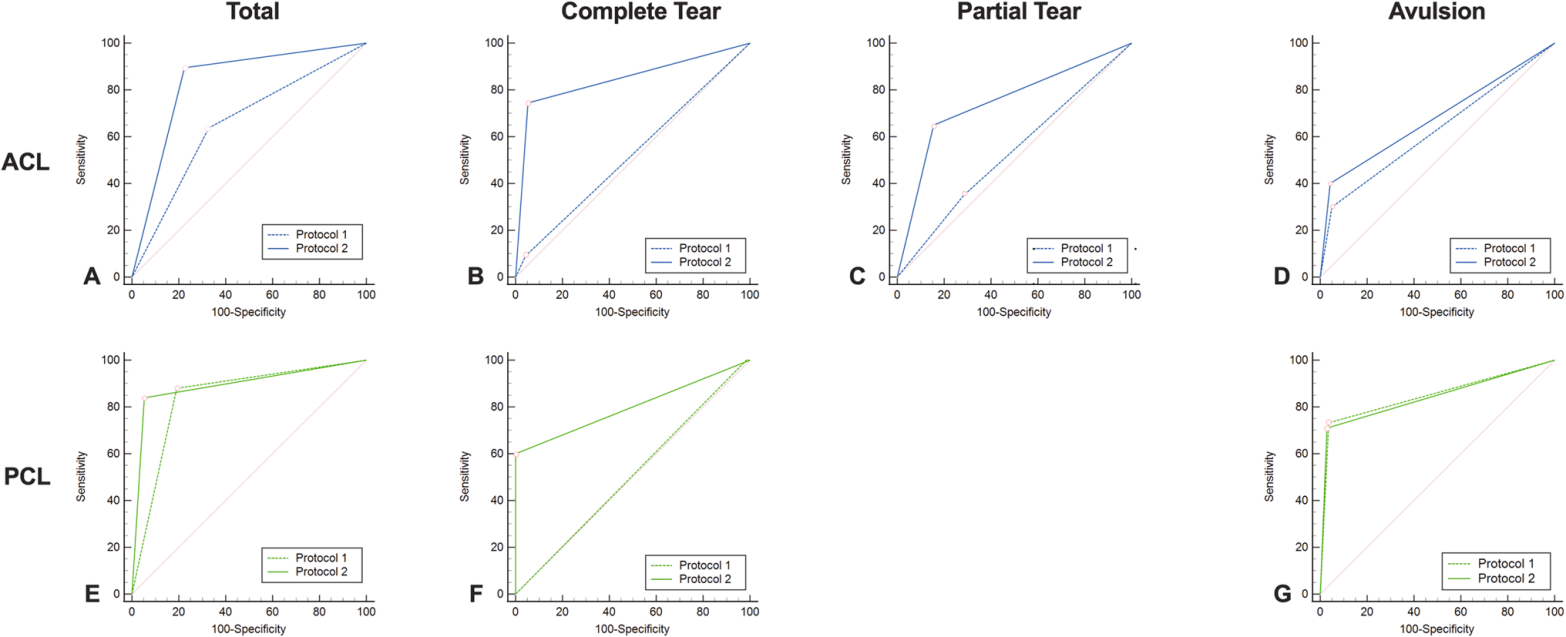
ROC curve analysis shows incremental value of protocol 2, including color-coded collagen reconstructions and grayscale CT (solid line) over protocol 1, comprising only standard grayscale CT (dotted line) for the depiction of all lesions (**A**, **E**), complete (**B**, **F**) and partial tears (**C**) of the cruciate ligaments. No incremental value was observed for avulsion fractures of the cruciate ligaments (**D**, **G**). Abbreviations: ROC, receiver-operating characteristic; ACL, anterior cruciate ligament; PCL, posterior cruciate ligament

Protocol 2, which included color-coded collagen reconstructions of the PCL, showed no significant difference for the detection of injury to the PCL compared to protocol 1 in overall sensitivity (42/50 [84%] vs. 44/50 [88%]), specificity (355/375 [95%] vs. 303/375 [81%]), PPV (42/62 [68%] vs. 44/116 [38%]), NPV (355/363 [98%] vs. 303/309 [98%]), accuracy (397/425 [93%] vs. 347/425 [82%]), and AUC (0.89 vs 0.84) (all comparisons, ΔAUC = 0.05, *p* = 0.20, Table 2). Inter-reader agreement was excellent for both protocols (*κ* = 0.87 for protocol 1 and *κ* = 0.90 for protocol 2), as well as between protocols (*κ* = 0.80). Notably, AUC increased significantly between protocol 1 and protocol 2 for complete PCL tears (0.80 vs 0.49, ΔAUC = 0.21, *p* = 0.02), but not for avulsions (ΔAUC = 0.01, *p* = 0.81, Table 2). Diagnostic accuracy parameters did not change significantly when arthroscopy was excluded as a reference standard. Agreement between MRI and protocol 2, including color-coded collagen reconstructions, was higher compared to agreement between MRI and protocol 1 for all injuries (Table 3). No partial PCL tears were observed in our study. An example case demonstrating improvement in the detection of PCL injury by color-coded collagen reconstruction is illustrated in Figs. 5 and 6.

### Diagnostic accuracy between different reference standards

The prevalence of injury to the cruciate ligaments was significantly increased in patients that received arthroscopy. In line, diagnostic accuracy was significantly higher when arthroscopy was available as the reference standard compared to MRI as reference standard (*p* < 0.001) (Supplementary Table 2).

### Diagnostic confidence, image quality, and image noise

The availability of color-coded collagen reconstructions increased the diagnostic confidence of all readers for the detection of injuries to the ACL (4.1 ± 0.6 vs. 2.7 ± 0.7, *p* < 0.001) and PCL (4.4 ± 0.7 vs. 3.0 ± 0.8, *p* < 0.001). Inter-reader agreement was moderate for protocol 1 (*κ* = 0.58 for ACL, *κ* = 0.52 for PCL) and good for protocol 2 (*κ* = 0.63 for ACL, *κ* = 0.65 for PCL). No significant differences were observed regarding the diagnostic confidence between less and more experienced readers (Table 4).

**Table 4:** Diagnostic confidence, image quality, and image noise of standard CT and color-coded collagen reconstructions for the ACL and PCL

	Diagnostic confidence ACL	Diagnostic confidence PCL	Image quality	Image noise
Grayscale images	2.7 ± 0.7 [2.6–2.7]	3.0 ± 0.8 [2.9–3.1]	2.8 ± 0.7 [2.7–2.8]	2.4 ± 0.8 [2.3–2.5]
Color-coded images	4.1 ± 0.6 [4.1–4.2]	4.4 ± 0.7 [4.3–4.5]	4.0 ± 0.6 [3.9–4.1]	3.8 ± 0.7 [3.8–3.9]
*p* value	< 0.001	< 0.001	< 0.001	< 0.001
*κ* grayscale	0.58 [0.42–0.70]	0.52 [0.33–0.66]	0.63 [0.49–0.74]	0.65 [0.51–0.75]
*κ* color-coding	0.63 [0.47–0.78]	0.65 [0.34–0.78]	0.64 [0.50–0.75]	0.72 [0.61–0.80]

*Abbreviations*: *ACL* anterior cruciate ligament, *PCL* posterior cruciate ligament

Numbers in square brackets are confidence intervals. Diagnostic confidence, image quality, image noise, and Fleiss’ *κ* of standard grayscale CT and color-coded collagen reconstructions

The image quality was rated with a mean score of 2.8 ± 0.7 for grayscale images and 4.0 ± 0.6 for color-coded collagen reconstructions, indicating that readers perceived image quality as superior in color-coded images (*p* < 0.001). Inter-reader agreement was comparable between grayscale and color-coded images (*κ* = 0.63 for grayscale, *κ* = 0.65 for color-coding).

Image noise was perceived lower in color-coded images compared to grayscale images (3.8 ± 0.7 vs. 2.4 ± 0.8, *p* < 0.001), and inter-reader agreement was similarly high (*κ* = 0.65 for grayscale, *κ* = 0.72 for color-coding).

## Discussion

A novel third-generation dual-source DECT postprocessing algorithm based on dedicated material decomposition facilitates color-coded collagen reconstructions of the cruciate ligaments. Our results show that these provide substantially higher overall diagnostic accuracy for the evaluation of complete and partial tears of the ACL compared to standard grayscale CT (AUC 0.85 vs. 0.52 and 0.75 vs. 0.53, respectively, all comparisons *p* < 0.001), and that ligamentous integrity of the cruciate ligaments can be accurately assessed in avulsion fractures.

Previous research demonstrated insufficient soft tissue contrast of conventional multi-detector CT (MDCT) for the assessment of the cruciate ligaments [11]. For second-generation dual-source DECT grayscale images, Peltola et al and Glazebrook et al showed an increased sensitivity (79% and 86%, respectively) for the detection of complete ACL tears [19, 20]. There are, however, significant methodical shortcomings in both studies, with a highly selected study population comprising fewer than 30 participants per study (18 patients, Peltola et al; 27 patients, Glazebrook et al) and the exclusion of patients with partial tears of the cruciate ligaments. In addition, both studies used an insufficient reference standard obtained without control imaging or blinding of the control readings, and dedicated analysis of color-coded collagen reconstructions was omitted or did not demonstrate incremental diagnostic value over grayscale CT [19, 20]. Since then, multiple studies have demonstrated improved material decomposition of third-generation dual-source DECT over second-generation dual-source DECT, which can be attributed to improved spectral separation by extending the disparate tube voltages up to 90 kV and tin-filtrated Sn150 kV, improvements in iterative image reconstruction, and advances in detector technology. Ultimately, these allow for a more detailed color-coded depiction of ligaments and tendons, which has been difficult to achieve before [21–27]. However, no studies have yet evaluated the potential of third-generation dual-source DECT and collagen mapping to improve the assessment of the cruciate ligaments. In our study, the diagnostic accuracy for the detection of injury to the ACL using grayscale images was significantly lower compared to Peltola et al and Glazebrook et al with a sensitivity of 64%, possibly due to a more heterogenous study population that comprised patients with complete tears, partial tears, and avulsion fractures. Furthermore, a more precise classification of the lesions was required from readers in our study, which remains challenging even in MRI. Notably, the availability of color-coded collagen reconstructions derived from a novel postprocessing algorithm based on dedicated material decomposition significantly increased the sensitivity of readouts to 90%, despite the heterogeneity of our study population and the more complex requirements for lesion classification. We observed no increase in diagnostic accuracy using color-coded collagen reconstructions for the identification of avulsion fractures of the cruciate ligaments. This result was expected, as grayscale images in a dedicated bone kernel were included in both protocols. Nonetheless, color-coded reconstructions facilitate assessing the integrity of the cruciate ligaments in avulsion fractures, as demonstrated by increased diagnostic accuracy and confidence of reporting radiologists for protocol 2 over protocol 1. The incremental diagnostic value of CT-based assessment of the cruciate ligaments using color-coded collagen reconstructions and the high agreement between color-coded reconstructions and MRI highlight the potential of DECT as an alternative imaging approach for patients with contraindications to MRI and in circumstances where MRI is not available. Additional applications of third-generation dual-source DECT, such as the depiction of bone marrow edema to identify acute fractures, further emphasize this role [16, 22, 26]. Reconstruction of color-coded collagen images from dual-energy CT took 3 min on average and is therefore applicable in daily clinical practice. In contrast to MRI, CT scans are readily available during on-call times, which can accelerate the diagnosis and treatment of acute ACL tears. By reducing the time of immobilization and the risk of subsequent injury to other soft tissue structures of the knee, accelerated treatment can improve patient outcomes and reduce the socioeconomic burden on health care systems and working environments [5–7].

This retrospective study has certain limitations we would like to address. First, because patients with fractures or a high probability of damage to the internal structures of the knee did not routinely receive MRI before surgical inspection, we used a mixed reference standard comprising MRI and/or arthroscopic inspection. Nevertheless, MRI represents the current imaging gold standard for cruciate ligament assessment besides arthroscopy; thus, we decided to include each patient undergoing at least one of both reference standards. Interestingly, the diagnostic accuracy was significantly increased when arthroscopy was available as a reference standard. We believe that this is not a technical issue but rather attributable to the fact that the prevalence of ligamentous injury was significantly higher in patients where arthroscopy was performed, and that the sustained injuries of these patients were more severe and readily picked up in imaging. This is underlined by the fact that the use of MRI as a single reference standard did not lead to a reduction of diagnostic accuracy. Nonetheless, further studies with larger patient’s cohorts should be performed to gain more insights into this finding. Second, we evaluated the incremental diagnostic value of third-generation dual-source DECT-derived color-coded collagen reconstructions over third-generation dual-source DECT grayscale images. Due to improved material differentiation, soft-tissue contrast has also improved significantly for standard grayscale images. Therefore, the incremental diagnostic value of collagen reconstructions over single-energy CT or previous generations of dual-source DECT could be underestimated from the results of this study. Last, the color-coded collagen reconstruction algorithm used in our study is vendor-specific; and therefore, the results of our study cannot be generalized, although all major vendors offer dual-energy CT.

In conclusion, our study shows that third-generation dual-source DECT color-coded collagen reconstructions substantially improve diagnostic accuracy for the detection of acute complete and partial tears of the cruciate ligaments of the knee compared to standard grayscale images, with inexperienced readers benefiting the most. Color-coded reconstructions demonstrate good agreement with MRI and achieve superior diagnostic confidence, image quality, and noise scores compared to grayscale CT for all readers. Therefore, we believe this algorithm may become a viable alternative for assessing the cruciate ligaments when MRI is unavailable, but dual-energy CT can be performed.

## Supplementary Information

Below is the link to the electronic supplementary material.

Supplementary file1 (PDF 209 kb) **Supplementary Table 1 ***Individual readings of diagnostic accuracy for the ACL and PCL. *Abbreviations: ACL: anterior cruciate ligament, PCL: posterior cruciate ligament, PPV: positive predictive value, NPV: negative predictive value, AUC: area under the curve. Numbers in square brackets are confidence intervals. Diagnostic accuracy of protocol 1 (standard grayscale CT) and protocol 2 (standard grayscale CT + color-coded collagen reconstructions) with MRI or arthroscopic inspection as the standard of reference. **Supplementary Table 2 ***Diagnostic accuracy of color-coded collagen reconstructions for the ACL and PCL for arthroscopic inspection as reference standard. *Abbreviations: ACL: anterior cruciate ligament, PCL: posterior cruciate ligament, PPV: positive predictive value, NPV: negative predictive value, AUC: area under the curve. Numbers in square brackets are confidence intervals. Diagnostic accuracy of protocol 2 (standard grayscale CT + color-coded collagen reconstructions) for arthroscopy as the reference standard. Prevalence of injury to the cruciate ligaments was higher in the group of patients with arthroscopic as the reference standard. Notably, for these patients the diagnostic accuracy was significantly increased compared to MRI, possibly due to more severe lesions that were easier to pick up in color-coded reconstructions.

## Abbreviations

ACL: Anterior cruciate ligament
AUC: Area under the curve
DECT: Dual-energy computed tomography
FOV: Field of view
MDCT: Multidetector computed tomography
NPV: Negative predictive value
PCL: Posterior cruciate ligament
PPV: Positive predictive value
ROC: Receiver operator characteristic
SD: Standard deviation

## Declarations

### Guarantor

The scientific guarantor of this publication is Prof. Dr. Thomas J. Vogl.

### Statistics and biometry

Neelam Lingwal of the Institute for Biostatistics of the Goethe Universität Frankfurt kindly provided statistical advice for this manuscript.

### Methodology

• retrospective

• diagnostic or prognostic study

• performed at one institution
