# Supplementary material for: Diagnostic value of DECT-based colored collagen maps for the assessment of cruciate ligaments in patients with acute trauma
Source: Eur Radiol. 2023 Mar 31;33(9):6339–50. doi: 10.1007/s00330-023-09558-4 (PMC10415420; doi:10.1007/s00330-023-09558-4)
Supplement: Supplementary file 1 — Supplementary file1 (PDF 209 kb) Supplementary Table 1 Individual readings of diagnostic accuracy for the ACL and PCL. Abbreviations: ACL: anterior cruciate ligament, PCL: posterior cruciate ligament, PPV: positive predictive value, NPV: negative predictive value, AUC: area under the curve. Numbers in square brackets are confidence intervals. Diagnostic accuracy of protocol 1 (standard grayscale CT) and protocol 2 (standard grayscale CT + color-coded collagen reconstructions) with MRI or arthroscopic inspection as the standard of reference. Supplementary Table 2 Diagnostic accuracy of color-coded collagen reconstructions for the ACL and PCL for arthroscopic inspection as reference standard. Abbreviations: ACL: anterior cruciate ligament, PCL: posterior cruciate ligament, PPV: positive predictive value, NPV: negative predictive value, AUC: area under the curve. Numbers in square brackets are confidence intervals. Diagnostic accuracy of protocol 2 (standard grayscale CT + color-coded collagen reconstructions) for arthroscopy as the reference standard. Prevalence of injury to the cruciate ligaments was higher in the group of patients with arthroscopic as the reference standard. Notably, for these patients the diagnostic accuracy was significantly increased compared to MRI, possibly due to more severe lesions that were easier to pick up in color-coded reconstructions. [file 330_2023_9558_MOESM1_ESM.pdf]

Supplementary Table 1

Individual readings of diagnostic accuracy for the ACL and PCL.

ACL Injury	Sensitivity	Specificity	PPV	NPV	Accuracy	AUC	*p*-value
Average							
Protocol 1	13.4/21 (64%) [82% – 95%]	43/64 (67%) [62% – 72%]	13.4/34.4 (39%) [34% – 44%]	43/50.6 (85%) [81% – 88%]	56.4/85 (66%) [62% – 71%]	0.65 [0.61 – 0.70]	<.001
Protocol 2	18.8/21 (90%) [82% – 95%]	49.6/64 (78%) [78% – 82%]	18.8/33.2 (57%) [51% – 62%]	49.6/51.8 (96%) [93% – 98%]	68.4/85 (80%) [76% – 84%]	0.84 [0.80 – 0.87]	<.001
Reader 1							
Protocol 1	12/21 (57%) [34% – 78%]	41/64 (64%) [51% – 76%]	12/35 (34%) [24% – 46%]	41/50 (82%) [73% – 89%]	53/85 (62%) [51% – 73%]	0.61 [0.49 – 0.71]	<.001
Protocol 2	20/21 (95%) [76% – 100%]	52/64 (81%) [70% – 90%]	20/32 (63%) [50% – 74%]	52/53 (98%) [88% – 100%]	72/85 (85%) [75% – 92%]	0.88 [0.80 – 0.94]	<.001
Reader 2							
Protocol 1	14/21 (67%) [43% – 85%]	43/64 (67%) [54% – 78%]	14/35 (40%) [30% – 51%]	43/50 (86%) [77% – 92%]	57/85 (67%) [56% – 77%]	0.67 [0.56 – 0.77]	0.36
Protocol 2	16/21 (76%) [53% – 92%]	46/64 (72%) [59% – 82%]	16/34 (47%) [36% – 58%]	46/51 (90%) [81% – 95%]	62/85 (73%) [62% – 82%]	0.74 [0.63 – 0.83]	0.36
Reader 3							
Protocol 1	12/21 (57%) [34% – 78%]	43/64 (67%) [54% – 78%]	12/33 (36%) [26% – 49%]	43/52 (83%) [74% – 89%]	55/85 (65%) [54% – 75%]	0.62 [0.51 – 0.73]	0.001
Protocol 2	20/21 (95%) [76% – 100%]	48/64 (75%) [63% – 85%]	20/36 (56%) [45% – 66%]	48/49 (98%) [88% – 100%]	68/85 (80%) [70% – 88%]	0.85 [0.76 – 0.92]	0.001
Reader 4							
Protocol 1	13/21 (62%) [38% – 82%]	42/64 (66%) [53% – 77%]	13/35 (37%) [27% – 49%]	42/50 (84%) [75% – 90%]	55/85 (65%) [54% – 75%]	0.64 [0.53 – 0.74]	0.004

Protocol 2	19/21 (90%) [70% – 99%]	50/64 (78%) [66% – 87%]	19/33 (58%) [46% – 69%]	50/52 (96%) [87% – 99%]	69/85 (81%) [71% – 89%]	0.84 [0.75 – 0.91]	0.004
Reader 5							
Protocol 1	16/21 (76%) [53% – 92%]	46/64 (72%) [59% – 82%]	16/34 (47%) [36% – 58%]	46/51 (90%) [81% – 95%]	62/85 (73%) [62% – 82%]	0.74 [0.63 – 0.83]	0.05
Protocol 2	19/21 (90%) [70% – 99%]	52/64 (81%) [70% – 90%]	19/31 (61%) [48% – 73%]	52/54 (96%) [87% – 99%]	71/85 (84%) [74% – 91%]	0.86 [0.77 – 0.93]	0.05
PCL Injury	Sensitivity	Specificity	PPV	NPV	Accuracy	AUC	*p*-value
Average							
Protocol 1	8.8/10 (88%) [76% – 95%]	60.6/75 (81%) [76% – 85%]	8.8/23.2 (38%) [33% – 44%]	60.6/61.8 (98%) [96% – 99%]	69.4/85 (82%) [78% – 85%]	0.84 [0.81 – 0.88]	0.20
Protocol 2	8.4/10 (84%) [71% – 93%]	71/75 (95%) [92% – 97%]	8.4/12.4 (68%) [57% – 77%]	71/72.6 (98%) [97% – 99%]	79.4/85 (93%) [91% – 96%]	0.89 [0.86 – 0.92]	0.20
Reader 1							
Protocol 1	8/10 (80%) [44% – 97%]	54/75 (72%) [60% – 82%]	8/29 (28%) [19% – 38%]	54/56 (96%) [89% – 99%]	62/85 (73%) [62% – 82%]	0.76 [0.66 – 0.85]	0.07
Protocol 2	9/10 (90%) [55% – 100%]	72/75 (96%) [89% – 99%]	9/12 (75%) [49% – 90%]	72/73 (99%) [92% – 100%]	81/85 (95%) [88% – 99%]	0.93 [0.85 – 0.97]	0.07
Reader 2							
Protocol 1	10/10 (100%) [69% – 100%]	63/75 (84%) [74% – 91%]	10/22 (45%) [33% – 58%]	63/63 (100%) [100% – 100%]	73/85 (86%) [77% – 92%]	0.92 [0.84 – 0.97]	0.11
Protocol 2	7/10 (70%) [35% – 93%]	67/75 (89%) [80% – 95%]	7/15 (47%) [29% – 65%]	67/70 (96%) [90% – 98%]	74/85 (87%) [78% – 93%]	0.80 [0.70 – 0.88]	0.11
Reader 3							
Protocol 1	10/10 (100%) [69% – 100%]	65/75 (87%) [77% – 93%]	10/20 (50%) [36% – 64%]	65/65 (100%) [100% – 100%]	75/85 (88%) [79% – 94%]	0.93 [0.86 – 0.98]	0.95
Protocol 2	9/10 (90%) [55% – 100%]	73/75 (97%) [91% – 100%]	9//11 (82%) [53% – 95%]	73/74 (99%) [92% – 100%]	82/85 (97%) [90% – 99%]	0.94 [0.86 – 0.98]	0.95
Reader 4							

Protocol 1	8/10 (80%) [44% – 97%]	53/75 (71%) [59% – 81%]	8/30 (27%) [19% – 37%]	53/55 (96%) [88% – 99%]	61/85 (72%) [61% – 81%]	0.75 [0.65 – 0.84]	0.24
Protocol 2	8/10 (80%) [44% – 97%]	72/75 (96%) [89% – 99%]	8/11 (73%) [46% – 89%]	72/74 (97%) [91% – 99%]	80/85 (94%) [87% – 98%]	0.88 [0.79 – 0.94]	0.24
Reader 5							
Protocol 1	8/10 (80%) [44% – 97%]	68/75 (91%) [82% – 96%]	8/15 (53%) [35% – 71%]	68/70 (97%) [91% – 99%]	76/85 (89%) [81% – 95%]	0.85 [0.76 – 0.92]	0.44
Protocol 2	9/10 (90%) [55% – 100%]	71/75 (90%) [55% – 100%]	9/13 (69%) [46% – 86%]	71/72 (99%) [92% – 100%]	80/85 (94%) [87% – 98%]	0.92 [0.85 – 0.97]	0.44

Supplementary Table 2

Diagnostic accuracy of color-coded collagen reconstructions for the ACL and PCL for arthroscopic inspection as reference standard.

Number of total examinations *or* *Number of lesions in modality*	Sensitivity	Specificity	PPV	NPV	Accuracy	AUC
Arthroscopic Inspection (n = 34)						
ACL (*n = 12*)	58/60 (97%) [89% – 100%]	102/105 (97 %) [92% – 99%]	58/61 (95%) [86% – 98%]	102/104 (98%) [93% – 100%]	160/165 (97%) [93% – 99%]	0.97 [0.93 – 0.99]
PCL (*n = 9*)	43/45 (96%) [85% – 100%]	115/120 (96%) [91% – 99%]	43/48 (90%) [78% – 95%]	115/117 (98%) [94% – 100%]	158/165 (96%) [92% – 98%]	0.96 [0.91 – 0.98]
